# The Shift of ERG B-Wave Induced by Hours' Dark Exposure in Rodents

**DOI:** 10.1371/journal.pone.0161010

**Published:** 2016-08-12

**Authors:** Dake Li, Qi Fang, Hongbo Yu

**Affiliations:** Vision Research Laboratory, School of Life Sciences, The State Key Laboratory of Medical Neurobiology, Collaborative Innovation Center for Brain Science, Fudan University, Shanghai, China; Carl von Ossietzky Universitat Oldenburg, GERMANY

## Abstract

**Purpose:**

Dark adaptation can induce a rapid functional shift in the retina, and after that, the retinal function is believed to remain stable during the continuous dark exposure. However, we found that electroretinograms (ERG) b-waves gradually shifted during 24 hours’ dark exposure in rodents. Detailed experiments were designed to explore this non-classical dark adaptation.

**Methods:**

In vivo ERG recording in adult and developing rodents after light manipulations.

**Results:**

We revealed a five-fold decrease in ERG b-waves in adult rats that were dark exposed for 24 hours. The ERG b-waves significantly increased within the first hour’s dark exposure, but after that decreased continuously and finally attained steady state after 1 day’s dark exposure. After 3 repetitive, 10 minutes’ light exposure, the dark exposed rats fully recovered. This recovery effect was eye-specific, and light exposure to one eye could not restore the ERGs in the non-exposed eye. The prolonged dark exposure-induced functional shift was also reflected in the down-regulation on the amplitude of intensity-ERG response curve, but the dynamic range of the responsive light intensity remained largely stable. Furthermore, the ERG b-wave shifts occurred in and beyond classical critical period, and in both rats and mice. Importantly, when ERG b-wave greatly shifted, the amplitude of ERG a-wave did not change significantly after the prolonged dark exposure.

**Conclusions:**

This rapid age-independent ERG change demonstrates a generally existing functional shift in the retina, which is at the entry level of visual system.

## Introduction

Eyes quickly regain their sensitivity to weak stimulus after being transferred from light to darkness [1]. This recovery process is well known as dark adaptation [1, 2]. Physiologically, the retinal response to the flash greatly increases within minutes to 2–3 hours (depending on the intensity of adapting light) as investigated by the electroretinogram (ERG) [3]. After that, the retinal function is assumed to become stable in complete darkness [4–7], and even during the critical period when visual plasticity is maximal, lots of previous studies have shown that prolonged dark exposure does not change retinal function or morphology in mammals [6, 8–11] (however, see hard-bone fishes [12]). Although circadian rhythm does modulate retinal responses in one day, it is primarily dependent on the internal clock instead of visual experience [13–16].

At the same time, a few recent studies have suggested that the retina may not be stable and irrelevant to previous visual experiences. For example, more than 20 days’ dark rearing after birth can change the visually evoked responses in the retinae of mice [17], and 12–20 days of light deprivation in adult cats can result in a significant decrease in the slope of the dark-adapted ERG b-wave amplitude-luminance function [18]. It suggests that the retina may be sensitive to long-term dark exposure [17–21]. However, it is still questionable whether the scotopic ERG can shift after short-term light manipulation such as hours’ dark exposure.

To investigate this question, we examined ERGs in adult and developing rodents (rats and mice) after one day of dark exposure. In particular, we strictly controlled the light during the entire rearing, transferring and recording periods. Our results demonstrated that the rodent ERG b-wave could shift within hours of dark exposure and beyond the classical critical period, and that this retinal functional shift was eye-specific and may originate from the retina. The results suggest that there is a general visual experience-dependent functional dynamics at the first stage of the visual pathway.

## Materials and Methods

### Ethic Statement

The animals were cared for and handled following the protocols approved by the Committee on the Ethics of Animal Experiments of the Fudan University and met the NIH guideline.

### Animals and light manipulation

The Sprague-Dawley (SD) rats and C57BL/6 mice (Shanghai Slac Laboratory Animal Co. Ltd.) were used in our experiments. Prior to each experiment, all animals received a cycle of 12 hours light/12 hours’ dark exposure with free access to food and water for one week, the average light intensity illuminating the cage during the subject day was 40 lux [21]. After that, the control animals were adapted to the dark for 30 minutes to attain a scotopic vision. The prolonged dark exposed (prolonged DE) animals were reared under complete darkness for 24 hours in a room with triple security of light, and then anesthetized to record ERGs. The dark exposed plus 3 repetitive, brief (10 minutes) light-exposed (prolonged DE + 3 LE) animals were treated with 24 hours’ prolonged dark exposure, then received 3 cycles of 10 minutes light exposure (0.16 log cd/m^2^) followed by 90 minutes of darkness (total of 300 minutes), and then anesthetized to record ERGs.

### ERG recordings

Light interference was eliminated before the ERG recording. The animals were dark-exposed, transferred and recorded under complete darkness. Anesthetization and fixation operations were performed with an infrared night vision device (Viking 1x24, Yukon Company), and the light intensity of this night device could not evoke any ERG responses (see S1 Fig).

ERG recordings. All ERGs were recorded under sufficient anesthesia. The rats were initially anesthetized by an intraperitoneal (i.p.) injection of avertin (250 mg/kg) and xylazine (10 mg/kg), and then anesthesia was maintained by an i.p. injection of avertin (60 mg/kg/h). Pupil dilation was achieved with a single drop of 1% tropicamide and 2.5% phenylephrine. One drop of 0.5% proparacaine hydrochloride was applied for corneal anesthesia. The rats were placed on a heating pad maintained at 38°C a silver ball electrode was placed in contact with the center of the cornea. The conjunction between the electrode and eye were covered with sterile lubricant eye gel (GenTeal Gel, NOVARTIS, contained hydroxypropyl methylcellulose). This gel is a thick jelly-like substance, which can keep the moist between the electrode and eye even at the end of our long-term recording. A stainless steel needle reference and ground electrode was placed on the animal’s forehead and tail, respectively.

The signals were amplified by 10,000. The data were acquired with a PowerLab 4/35 (ADInstruments, Bella Vista, NSW, Australia) data acquisition system (sampling rate: 1000Hz) with a band-pass filtered between 0.3Hz to 500 Hz and analyzed with custom software written in Matlab (The MathWorks, Natick, MA).

The white test flashes and exposure lights were generated by a Ganzfield type of LED, and the light intensity was controlled by changing the output voltage with the PowerLab system and calibrated using a photometer (Model IL1700; International Light, Newburyport, MA, USA).

Each ERG trial was 1200 ms, starting with a 200 ms baseline (in the dark), followed by a 50 ms flash (0.16 log cd/m^2^ which equivalent to -1.14 log cd*s/m^2^) and 950 ms in the dark.

To increase the signal-to-noise ratio, 9 trials were consecutively recorded and averaged for the dark-adapted ERGs. Due to low light intensity, the ERGs between trials did not decay significantly. The pre-flash signal was obtained as a baseline, and the ERG b-wave was then measured from the baseline to maximum value within 250 ms after the flash onset. The peak latency time was measured from the flash onset to the time point with a maximum value.

For ERG a-wave test, a stimulus with a stronger light intensity (0.82 log cd*s/m^2^, 50ms) was used to induce an ERG response with an a-wave. Each ERG trial was 1200 ms, starting with a 200 ms baseline (in the dark), followed by a 50 ms flash and 950 ms in the dark. The ERG waves of 3 trials (recorded with 50-second interval) were averaged for the dark-adapted ERGs.

In the Time Course of ERG Suppression experiments, normally reared animals (12 hours’ light/12 hours’ dark) were anesthetized and placed in complete darkness for 7 hours, during which their ERGs were recorded every 30 minutes.

In the Brief Light Exposure on the Prolonged Dark Exposure Rat experiment, the animals were reared in darkness for 24 hours, then anesthetized, and scotopic ERGs were recorded every 5 minutes. After that, the rats were treated with 10 minutes (0.16 log cd/m^2^) of brief light exposure, and then scotopic ERGs were continuously recorded in complete darkness. It is notable that exposure light with a different intensity could also trigger the similar recovery effect (See S2 Fig).

In the Monocular Light Exposure experiment, the animals were treated with one day of dark exposure. Using a night vision device, both the recording electrodes and LED lights were covered with opaque masks and were placed over the subjects’ eyes. A blackout cloth was used to cover the area where the mask fit on the rat’s face to minimize light exposure on the other eye. First, scotopic ERGs were recorded simultaneously for both eyes. Then, for monocular light exposure, LED light was applied to one eye, whereas the other eye did not receive light exposure. Thus, for each animal, the non-exposed eye served as the control. After monocular light exposure, the LED light was turned off, and binocular ERG recordings were obtained.

For the Light Intensity-ERG Response curve, the animals were stimulated with 8 lights of various intensities (-3.53, -2.91, -2.64, -2.23, -1.82, -1.54, -1.14, and -0.60 log cd*s/m^2^).

### Statistical Analysis

Analyses were performed using MATLAB. The results are represented as the mean± SEM. p values of <0.05 were considered statistically significant.

## Results

### Prolonged dark exposure-induced suppression of ERGs and restoration by subsequent light exposures

**Fig 1:**
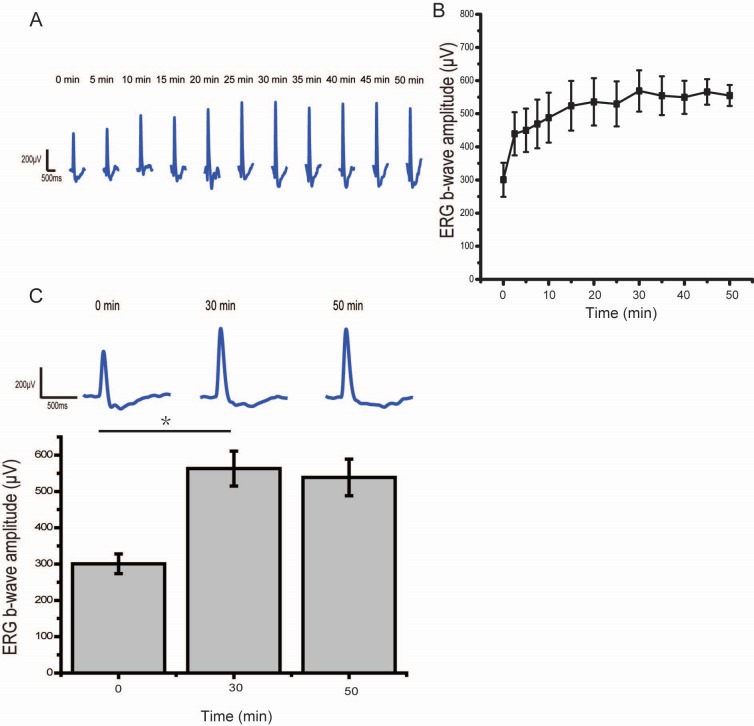
Classical dark adaptation in ERG responses. Normally reared rats were put into a dark environment, and scotopic ERGs response to a 50 ms flash (-1.14log cd*s/m^2^) were recorded within the initial 50 minutes dark adaptation. (A) ERGs across time from one rat. (B)The Statistics of the b-wave amplitudes at each recording time (n = 4, error bars, ± SEM). (C) The scotopic ERG amplitudes at 3 time points (0 minutes, 30 minutes, 50 minutes) after the onset of dark exposure (n = 4, error bars, ± SEM).

Dark adaptation is well described as the rapid enhancement of the retinal response during a sudden transition from light to darkness, and ERG amplitude significantly increases within the initial 30 minutes of darkness. Before hours’ dark exposure, we first confirmed this rapid classical dark adaptation effect. A typical dark-adapted ERG evoked by a dim flash consists mainly of the b-wave, which comes from the cumulative depolarization of rod DBCs [22, 23]. In our experiments, rats were put into a dark environment, and scotopic ERGs to a 50 ms flash (-1.14 log cd*s/m^2^), which could evoke robust b-wave responses [19, 21], were recorded within the initial 50 minutes. As expected, the ERG b-waves showed a rapid enhancement within the first 10 minutes of dark adaptation, and after 30 minutes’ dark exposure, the scotopic ERG started to reach a stable state (Fig 1A–1C, 562.9 ± 48.1 μV recorded after 30 minutes of dark adaptation vs 538.6 ± 50.5 μV recorded after 50 minutes of dark adaptation, n = 4, paired t test, t = 1.34, p = 0.27>0.05), which is consistent with previous reports [24, 25].

**Fig 2:**
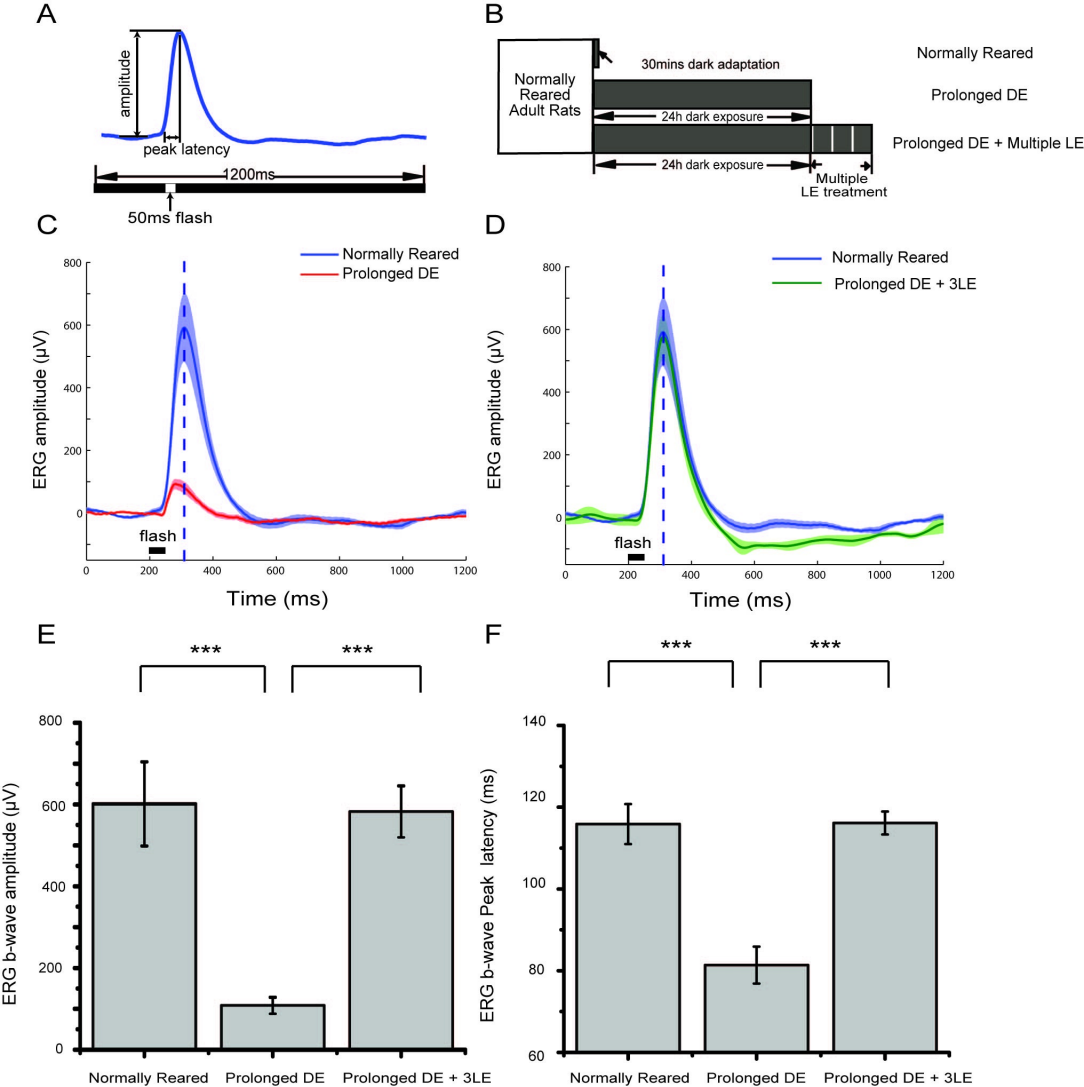
Prolonged dark exposure induced shift in ERG response and its restoration by subsequent light exposure. (A) ERG amplitude and peak latency measurements. (B) The preparation prior to ERG recording of three groups of rats. (C) A comparison of b-wave responses to a -1.14 log cd*s/m2 flash between normally reared (blue, n = 8) and dark prolonged rats (red, n = 8) showed a significant b-wave suppression. The mean response is represented by the solid line, and SEM is shown as the shadow. Dotted line shows the peak latency in normally reared rats. (D) A comparison of b-wave responses response to a 50 ms flash (-1.14log cd*s/m^2^) between normally reared rats (blue, n = 8) and prolonged dark exposed plus 3 repetitive, brief (10 minutes) light exposed rats (green, n = 8). Symbols are identical to those in C. (E) The b-wave amplitudes of the three groups (normally reared rats, n = 8; Prolonged DE rats, n = 8; Prolonged DE+3 LE rats, n = 8). (F) The statistics of the b-wave peak latencies of the three groups, samples, and symbols are identical to those in E (*** p<0.001, t test, error bars, ±SEM).

However, in the prolonged dark exposed (Prolonged DE) rats, in which the animals were continuously dark exposed for 24 hours, then anesthetized with the help of an infrared night device, and recorded in complete darkness (see Methods), the amplitude of the ERG b-wave evoked by an identical flash (50 ms, -1.14 log cd*s/m^2^) was much lower (Fig 2C, red line) than that of the normally reared group (anesthetized and recorded after 30 minutes of dark adaptation, Fig 2C, blue line). Interestingly, for prolonged dark exposed rats group, 3 repetitive, brief light exposure (LE), which included 10 minutes of light exposure (0.16 log cd/m^2^) followed by 90 minutes of dark exposure for each trial (Fig 2B,DE + 3LE), were sufficient to restore the ERG b-wave amplitude (Fig 2D, green line). Although the general shape of the ERG did not change, the peak latency of the b-wave (Fig 2C, dotted line) was shortened after 24 hours of dark exposure, and again was restored by 3 repetitive, brief (10 minutes) subsequent light exposures (Fig 2D). Across groups (Fig 2E and 2F), amplitude reduction (601.4 ± 102.9 μV in the control n = 8, vs 107.6 ± 20.8 μV in the Prolonged DE group, n = 8, t test, t = 4.70, p<0.001) and peak latency shift (115.9 ± 4.9 ms in the control, n = 8, vs 81.4 ± 4.5 ms in the Prolonged DE group, n = 8, t test, t = 5.19, p<0.001) were all statistically significant, except for the normally reared versus recovery (Prolonged DE + 3LE) groups (b-waves amplitude: 582.0 ± 63.4 μV, t = 0.16, p = 0.87>0.05, n = 8, t test; b-waves peak latency: 116.1 ± 2.8ms, t = 0.04, p = 0.97>0.05, n = 8, t test).

It is also notable that even in the control rats, ERG b-wave amplitudes did vary a lot from case to case (562.9 ± 48.1 μV in Fig 1C vs 601.4 ± 102.9 μV in Fig 2C, different mean values, but p = 0.14, ANOVA, F = 2.48, due to large SEMs), which could be consistently found in previous ERG studies [3, 26]. Continuous recordings and subsequent comparison in the same rat may help to control the variability across animals. Furthermore, ERGs in the above three groups of rats were all recorded under sufficient anesthesia to exclude its possible influence, but the manipulations of dark exposure and light exposure were performed in awake rats. It is also desirable to investigate the ERG shifts induced by light manipulations during the anesthetized state.

### The time course of ERG suppression during prolonged dark exposure

**Fig 3:**
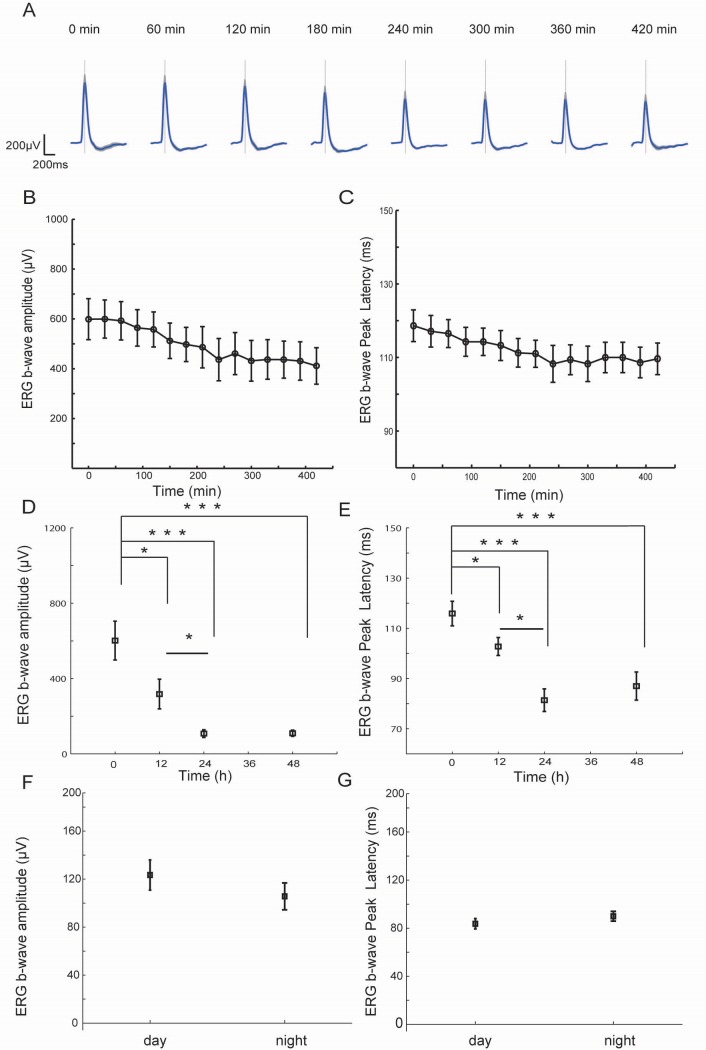
ERG amplitude and peak latency gradually decreased after the continuous dark exposure. (A) ERGs evoked by a 50 ms flash (-1.14log cd*s/m^2^) recorded during continuous dark exposure for 7 hours. Time 0 was set as 30 minutes after the onset of dark exposure. Gray lines are the peak latency at time 0. The solid blue lines are mean responses (n = 8), and the gray shadows represent SEM. (B) The statistics of the b-wave amplitudes at each recording time (n = 8, error bars, ± SEM). (C) The b-wave peak latencies recorded at different time points during continuous dark exposure. (D) The mean b-wave amplitudes of ERG response to a 50 ms flash (-1.14log cd*s/m^2^) of rats dark exposed for 0, 12, 24 and 48 hours (n = 8 for each group). The mean values of each group are illustrated by black squares (* p<0.05, *** p<0.001, t test). (E) The b-wave peak latency recordings at the identical time points in D. (* p<0.05, *** p<0.001. t test). (F)The b-wave amplitudes of the dark exposed rats (n = 10) to a 50 ms flash (-1.14log cd*s/m^2^) at day (6:00–18:00) and night (18:00–6:00) time period. (G)The ERG b-wave peak latencies in the above-mentioned two groups. The short vertical solid bars denote SEM in B-G.

To investigate how ERG suppression develops in the same anesthetized animal, we anesthetized a normally reared rat and continuously recorded ERGs for 7 hours in complete darkness. We set 30 minutes after the onset of dark exposure as time point 0, since at that time, the classical dark adaptation effect reached the steady state in our experiments (Fig 1). After that, both the amplitude and peak latency of the ERG b-wave decreased progressively over 420 minutes (Fig 3A–3C), which is consistent with the decline tendency suggested by acute one time-point data at 24 hours in Fig 2.

We then compared 4 groups of rats’ ERG b-waves which were recorded at the time point of 0, 12, 24 and 48 hours (n = 8 for each group) of dark exposure respectively, since it was hard to continuously record ERGs in the same rat under long-term stable anesthesia. The ERG b-wave amplitudes of the 12 hours dark exposed rats showed a significant decrease compared to the normally reared rats (Fig 3D, 601.4 ± 102.9 μV vs 317.7 ± 78.5 μV after 12 hours of dark exposure, t test, t = 2.191, p = 0.045<0.05). Also, the peak latency of the b-wave decreased significantly from 115.9 ± 4.9ms (time point 0) to 102.8 ± 3.6 ms after 12 hours of dark exposure (t test, t = 2.172, p = 0.048<0.05).

This 12 hours dark exposure data were further compared to the 24 hours dark exposed group, the b-wave amplitude decreased to 107.6 ± 20.8 μV (t test, t = 2.585, p = 0.022<0.05, Fig 3D), and peak latency further decreased to 81.4 ± 4.5 ms after 24 hours of dark exposure (t test, t = 3.728, p = 0.002<0.05, Fig 3E). The decreases in both amplitude and peak latency of the b-waves in the prolonged dark exposed rats were progressive and long-lasting in the first 24 hours of dark exposure.

However, both the amplitude and peak latency showed no further decline between the 24- and 48 hours dark exposed animals’ ERGs (48 hours group’s amplitude, 109.4 ± 16.7 μV, compared with 24 hours group, t = 0.07, p = 0.94>0.05, t test; 48 hours group’s peak latency, 87.0 ± 5.6 ms, compared with 24 hours group, t = 0.44, p = 0.78>0.05, t test), indicating that after 24 hours of dark exposure, the ERG b-wave reached a steady state (Fig 3D and 3E).

ERGs can fluctuate according to the internal circadian rhythm, which is around 24 hours, [16, 27, 28]. However, this fluctuation is different from our data shown in Fig 3D, which demonstrates an obviously progressive and one-way decline over 24 hours. To further examine the possible influence of internal circadian rhythms, ERG recording trials were repeated at projected day and night time intervals. No difference was observed between the ERG b-waves in the 24 hours dark exposed rats (n = 10) tested at different time points (Fig 3F, amplitude at projected day time: 123.4 ± 12.6 μV, night: 105.7 ± 11.2 μV, t test, t = 1.05, p = 0.30>0.05; Fig 3G, peak latency at projected day time: 83.7 ± 4.3 ms, night: 89.9 ± 4.1 ms, t test, t = 1.04, p = 0.31>0.05), demonstrating that the ERG shifts we observed was not influenced by the internal circadian rhythm.

### Brief light exposure triggers the recovery of ERG in prolonged dark exposed rats with eye specificity

**Fig 4:**
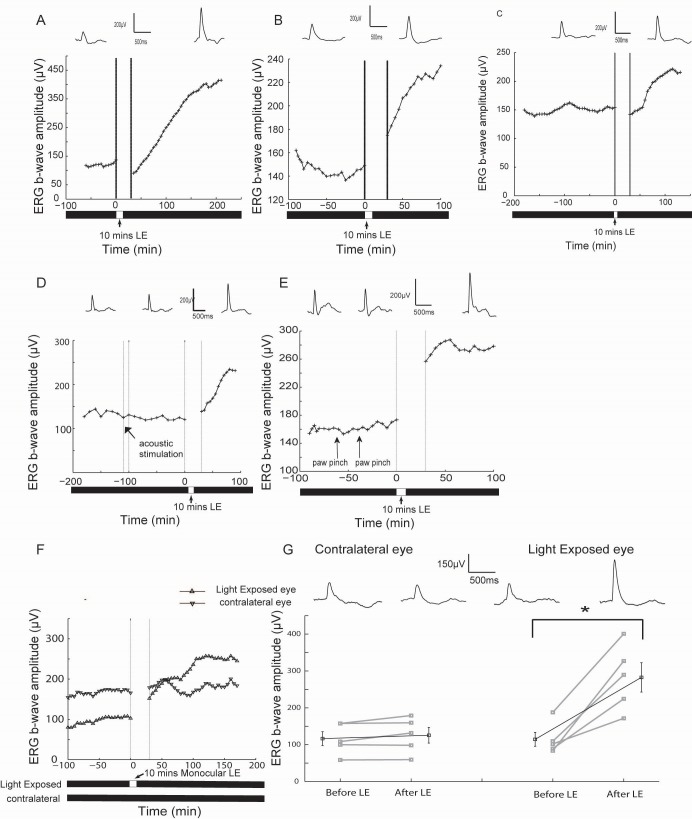
Prolonged dark exposure induced suppression was restored by a subsequent light exposure with eye specificity. (A-C) Three typical cases showing that ERG b-wave amplitudes of the prolonged dark exposed (Prolonged DE) rats after the 10 minutes light exposure (LE). Typical ERG curves before and after light exposure were presented on top of each panel. (D) The ERGs of a Prolonged DE rat after an acoustic stimulation and subsequent 10 minutes light exposure. The acoustic white noise (100dB) lasted for 10 minutes (arrow). (E) The ERGs of a prolonged DE rat after paw pinches and subsequent 10 minutes light exposure. The rat’s paws were pinched by forceps (arrows). (F) Incremental effects of monocular light exposure on the ERGs of dark exposed rats. (G) Binocular ERG b-waves recorded before and after 10 minutes monocular light exposure in dark exposed rats. The mean values of each group are illustrated by black squares, and the gray squares show individual cases (* p<0.05, paired t test). The short vertical solid bars denote SEM. The ERG in this figure were all evoked by a 50 ms white flash (-1.14log cd*s/m^2^).

As Fig 2 showed, 3 repetitive, brief (10 minutes) light exposures in the 24 hours dark exposed rats could restore the ERG reduction. During this experiment, the ERG was recorded during anesthesia, but the light exposures were manipulated in awake animals. Next, ERG restoration by brief light exposure was continuously monitored in the same anesthetized animal. In rats dark exposed (in awake state) for one day, we anesthetized the rats in complete darkness, recorded baseline ERGs and then provided 10 minutes’ light exposure (0.16 log cd/m^2^) to the rats. Since a sudden light was exposed to the prolonged dark exposed rats, a rapid light adaptation occurred first and 30 minutes’ dark adaptation was needed to restore the scotopic vision. We waited 30 minutes to re-examine the ERGs. It seemed that this brief light exposure could progressively recover b-wave reductions elicited by the prior dark exposure (Fig 4A). Importantly, restoration was closely related to the starting point of light exposure, while the prior test flashes (to evoke ERGs) or the anesthetized time appeared irrelevant to the restoration process (Fig 4A–4C, with the baseline recording time varying from 60 to 240 minutes, recorded at various time intervals until 10 minutes of light exposure was introduced). It suggested that 10 minutes of light exposure enhanced ERG, but multiple 50ms test flashes in the pre-exposure period did not.

We then investigated the origin of this light-induced restoration in the nervous system of dark exposed rats. At first, we examined the possible influence of inducing an autonomous startle response, since a sudden light exposure after 24 hours of complete darkness might cause a startle response. Acoustic and pinch stimuli were thus applied to the dark exposed rats before a light exposure was used, and no difference in b-wave amplitudes was observed after either stimulus set (Fig 4D and 4E, arrows). As a control, the subsequent 10 minutes’ light exposure triggered significant recovery (Fig 4D and 4E). Taken together, it appears that restoration is elicited by light and thus occurs via the excitation of the visual pathway.

Except for the retina, most visual pathway regions receive binocular inputs. To examine whether the retinal functional shift we observed originated from the retina, we measured the restoration effect on the ERGs of both eyes when only one eye was exposed to light (see Methods). After a 10 minutes monocular light exposure in the prolonged dark exposed rats, the ERG b-wave amplitude of the light exposed eye started to recover, whereas the b-wave of the non-light exposed eye remained unchanged (Fig 4F). The b-wave amplitudes of 5 prolonged dark exposed rats before and 130 minutes after the onset of monocular light exposure showed that restoration only occurred in exposed eyes (Fig 4G): the b-wave amplitude shifted from 114.5 ± 18.9 μV to 282.6 ± 39.8 μV in the light exposed eye (t = 5.503, p = 0.005<0.05, n = 5, paired t test), but not in non-light exposed eyes (from 116.4 ± 18.8 μV to 125.6 ± 21.3 μV, t = 1.693, p = 0.165>0.05, n = 5, paired t test). The ERG comparison between different eyes of the same animal demonstrated an obvious eye-specific retinal functional shift, thus suggested a local retinal mechanism.

It is notable although brief light exposure could trigger restoration process, the amplitude of restoration varied across animals. Furthermore, compared with Fig 2D when multiple (3) light exposures and enough interleaved darkness time (three 90 minutes) were provided, the restoration in Fig 4 was partial and slow. It suggested that the restoration could be rapidly triggered but developed in a slower pace.

### ERG shifts in and beyond the classical critical period and in both rats and mice

In the primary visual cortex, most of the functional plasticity has been reported in the classical critical period, which is 21–35 days after birth in rats [29]. During this developmental stage, rodents show strong visual plasticity, i.e., visual experience-dependent functional shift during this stage is most evident. However, recent studies also demonstrated a weaker but significant plasticity in the adult rodent primary visual cortex [30–32]. It is thus interesting to investigate the retinal functional shift we observed in this study in and beyond the classical critical period.

**Fig 5:**
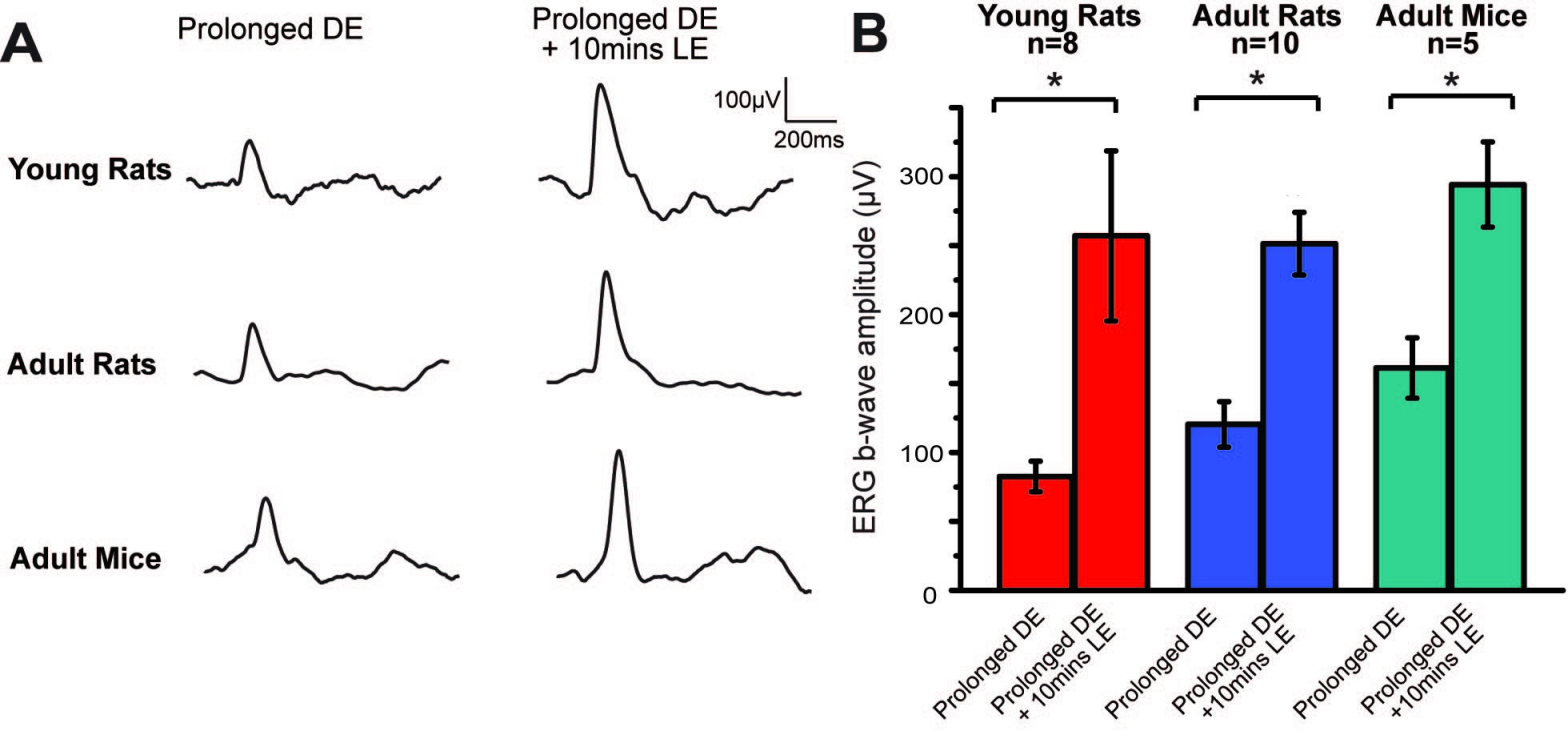
ERG shifts in and beyond the classical critical period and in both rats and mice. (A) ERG b-waves response to a 50 ms flash (-1.14log cd*s/m^2^) recorded from one animal in each group before and after 10 minutes light exposure. (B) Restoration effects in ERG b-wave amplitudes from the three groups before and after 10 minutes of light exposure (* p<0.05, all paired t test). Short vertical solid bars denote SEM.

Three groups of animals, including young rats (aged 3–4 weeks, considered to be in the critical period, n = 8), adult rats (aged 7–8 weeks, n = 12), and adult mice (aged 7–8 weeks, n = 5) were dark exposed for 24 hours. A 10 minutes’ light exposure was then utilized to trigger the restoration process. Fig 5A demonstrates that 130 minutes after the onset of 10 minutes’ light exposure, the young rats and adult mice underwent a significant enhancement in ERG b-wave amplitudes similar to adult rats, and that this result is consistent across animals (Fig 5B. young rats: 82.6 ± 11.1 and 257.1 ± 61.6 μV, before and after, respectively, t = 2.84 p = 0.02<0.05; adult rats: 120.4 ± 16.5 and 251.2 ± 22.7 μV, t = 7.023, p<0.001; adult mice: 161.3 ± 21.8 and 294.3 ± 30.9 μV, t = 6.51, p = 0.002<0.05, all paired t test). These results demonstrated that the retinal functional shift we found was present both in and beyond the critical period of rodents. Taking account of the possible retinal contribution, it may help to elucidate the functional dynamics of the visual cortex in young and adult rodents.

### Visual experience-dependent light intensity-ERG response curve

The ERG amplitude varies with the intensity of the test flash, and the light intensity-ERG response curve is sensitive to the available rhodopsins in the outer segments of photoreceptors and other neural factors [3]. In the above tests, a fixed intensity of the flash was used to evaluate ERG b-wave shift, and one may argue that the b-wave amplitude reduction after the prolonged dark exposure was due to the rightward shift of the intensity-response curve. It is thus important to examine the visual experience-dependent ERG shift using various light intensities.

**Fig 6:**
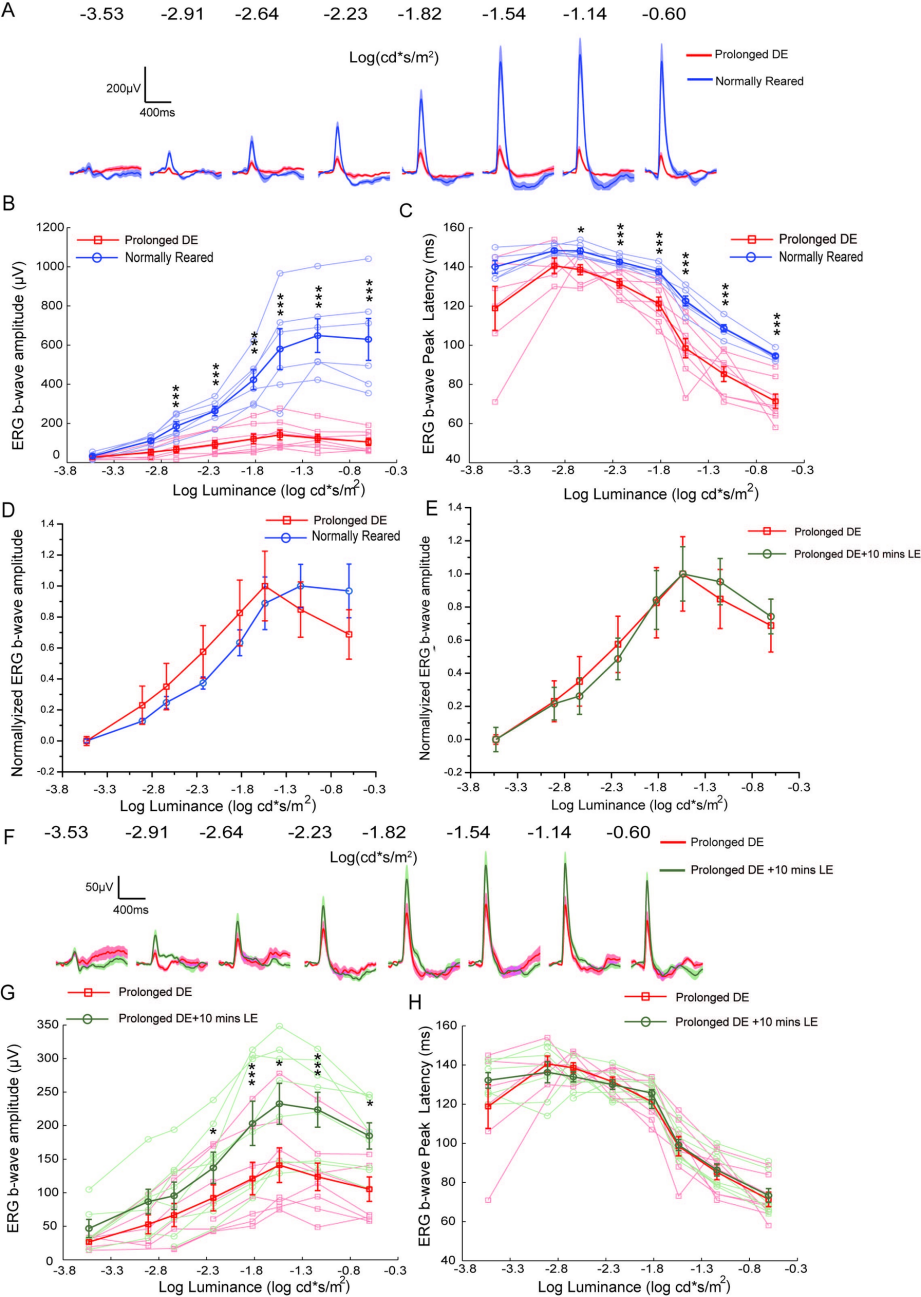
The shift of light intensity-ERG response curves after light manipulations. (A) Mean ERG b-waves of the 24 hours dark exposed (DE) rats (n = 8, red) and normally reared rats (n = 6, blue), SEM is shown as the shadow. (B) Light intensity-ERG amplitude curves to the 8 light intensities for the 24 hours dark exposed (prolonged DE) (n = 8, red) and normally reared rats (n = 6, blue). Mean (dark trace) and individual (light trace) were shown. * p<0.05, *** p<0.001, ANOVA. (C) Light intensity-peak latency curves of the b-waves in the 24 hours dark exposed rats (n = 8, red) and the normally reared rat eyes (n = 6, blue). (D) Normalized light intensity-ERG amplitude curves of the 24 hours dark exposed (n = 8) and normally reared rats (n = 6). (E) Normalized light intensity- ERG amplitude curves of the 24 hours dark exposed rats (n = 8) before (red squares) and after the 10 minutes light exposure (green circles). (F) Mean ERGs of the 24 hours dark exposed rats obtained before the 10 minutes light exposure (n = 8, red) and 130 minutes after the exposure (n = 8, green). (G) Light intensity-ERG amplitude curves of 24 hours dark exposed rats (n = 8) before (red squares) and after the 10 minutes light exposure (green circles). * p<0.05, *** p<0.001, paired t test. (H) Light intensity-peak latency curves of 24 hours dark exposed rats (n = 8) before (red squares) and after the 10 minutes light exposure (green circles). Vertical short bars denote SEM in B-D and F-H.

The rats were tested using 8 different light intensities (from -3.53 to -0.60 log cd*s/m^2^), which could effectively evoke a linear intensity-response curves of b-waves in the low intensity range, as described previously [19, 21]. When the test flash intensity increased, the b-wave amplitudes progressively increased (Fig 6A and 6B, blue, normally reared rats) and peak latency decreased (Fig 6C, blue), as expected [21, 26, 33]. Importantly, the ERG b-wave amplitudes in the one-day dark exposed rats were consistently lower than those in the normally reared group for all flash intensities (red vs blue traces in Fig 6A). Compared with the prolonged dark exposed rats (from 26.6 ± 3.3 μV, induced by -3.53log cd*s/m^2^ light intensity to 141.1 ± 25.7μV, n = 8, induced by -1.14 log cd*s/m^2^ light intensity, Fig 6B, red squares), the b-wave amplitudes for normally reared rats (from 32.8 ± 7.5 μV, induced by -3.53 log*s cd/m^2^ light intensity to 648.8 ±86.3 μV, n = 6, induced by-1.14 log cd*s/m^2^ light intensity, Fig 6B, blue circles) were significantly higher (Fig 6B, asterisks denote statistical significance, ANOVA). The peak latencies in normally reared rats were significantly greater than those in prolonged dark exposed rats when light intensity exceeded -2.64 log cd*s/m^2^ (Fig 6C, asterisks denote statistical significance). It is notable that in normal animals, the peak latencies increased when b-wave amplitudes decreased. In the dark exposed rats, the b-wave amplitudes were significantly lower than those in normal rats, but the peak latencies were shorter, suggesting a different mechanism for the dark exposed animals. Furthermore, after normalization (minimal amplitude to be 0 and maximal amplitude to be 1, respectively for normal and dark exposed rats), the intensity-response curves of the normal and prolonged dark exposed groups were similar, which demonstrated a largely unchanged dynamic range of light intensities (Fig 6D) and suggested a linear amplification mechanism.

In the recovery protocol, the prolonged dark exposed rats were exposed to 10 minutes’ light and recorded 130 minutes later, and we found that this brief light exposure enhanced the ERG b-wave amplitudes at nearly all intensities (Fig 6F, red vs green traces). The b-wave amplitudes after the light exposure (from 46.7 ± 13.6 μV, induced by -3.53 log cd*s/m^2^ light intensity to 232.6 ± 30.5μV, n = 8, induced by -1.14 log cd*s/m^2^ light intensity, Fig 6G, green circles) appeared higher than those before brief light exposure in prolonged dark exposed rats (from 26.6 ± 3.3 μV, induced by -3.53 log cd*s/m^2^ light intensity to 141.1 ± 25.7μV, n = 8, induced by -1.14 log cd*s/m^2^ light intensity, Fig 6G, red squares; Asterisks denote statistical significance, paired t test). However, there were no obvious changes in peak latency (from 140.6 ± 4.0 to 71.4 ± 3.7 ms for prolonged dark exposed rats and from 136.3 ± 5.4 to 73.4 ± 3.5 ms for light-exposed rats, Fig 6H). This result may be due to the partial recovery effect induced by only one session of 10 minutes light exposure (compared to the 3 repetitive 10 minutes LE shown in Fig 2D–2F). Similarly, the normalized intensity-response curves of dark exposed and light-exposed rats were nearly identical (Fig 6E), which again suggested an unchanged dynamic range of light intensities, with a linear amplification in response amplitude.

### Prolonged dark exposure-induced suppression of ERG b-waves without change of a-waves

**Fig 7:**
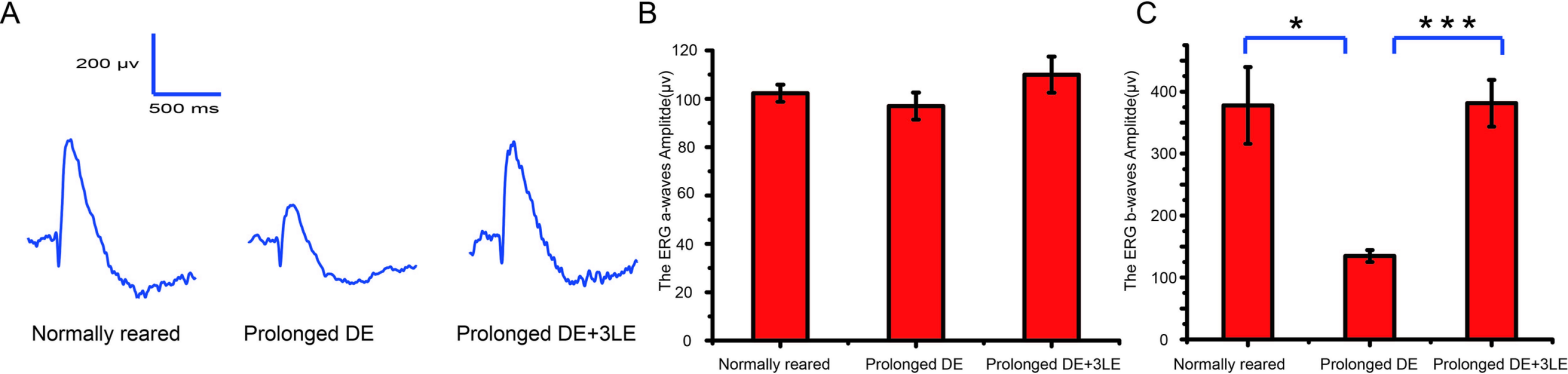
The ERG a-wave and b-wave after prolonged dark exposure in rats. (A)Typical cases of ERG waves to the 0.82 log cd*s/m^2^ light stimulus in three groups of rats (Left to right: normally reared group, prolonged dark exposed group, recovery group which was the prolonged dark exposed group followed by 3 repetitive, brief (10 minutes) light exposure). (B) The statistics of the a-wave amplitudes in three groups (n = 4). (c) The statistics of the b-wave amplitudes in three groups (n = 4). (* p<0.05, t test, error bars ±SEM).

To investigate whether the photoreceptors were involved in the ERG b-wave reduction after prolonged dark exposure, a stronger light intensity (0.82 log cd*s/m^2^, 50ms) were applied to induce the ERG a-waves and b-waves in three groups of rats (normally reared group, prolonged dark exposed group, recovery group which was the prolonged dark exposed group followed by 3 repetitive, 10 minutes light exposure, Fig 7A). As expected, the amplitudes of ERG b-waves significantly decreased after this prolonged dark exposure (Fig 7C, 134.7±9.6 μV in the prolonged dark exposed group, n = 4, vs 377.9 ± 62.0 μV in the normally reared group, n = 4, t = 3.87, p = 0.008<0.05, t test) and were restored by subsequent 10 minutes of light exposure (Fig 7C, 134.7±9.6 μV in the prolonged dark exposed group, n = 4 vs 381.3± 37.7 μV in the recovery group, n = 4, t = 6.34, p<0.001, t test). However, the ERG a-wave recorded in the prolonged dark exposed group (97.0 ± 5.6μV, n = 4) showed no significant decrease compared with the normally reared group (102.3± 3.5 μV, n = 4, p = 0.45>0.05, t test) and recovery group (110.0 ± 7.5 μV, n = 4, p = 0.21>0.05). This result suggested that the function of photoreceptors were not modified by this prolonged dark exposure.

## Discussion

The major results of the present study was that 24 hours dark exposure and subsequent brief light exposure significantly modulated the dim light evoked ERG b-waves of the retina; this functional shift was eye-specific, rapid, age-independent and occurred in both rats and mice. This 24 hours dark exposure-induced functional shift (5-fold ERG amplitude decrease, peak latency decrease) was opposite to the classical dark adaptation (rapid amplitude increase and peak latency increase within the initial 1 hour), and it is also different from the prediction based on the standard light intensity-ERG curve (the peak latency increases when ERG b-wave amplitude decreases [21, 26, 33], also see Fig 6C, blue trace), suggesting a new underlying mechanism. The a-waves evoked by stronger light stimuli showed the ERG b-wave decreased with the prolonged dark exposure without a significant reduction of the a-wave amplitude. This result suggests this ERG b-wave depression may not originate from the functional shift of photoreceptors. To the best of our knowledge, this is the first study to report that the retina, as the first stage of the visual system, can modulate its function following hours of dark exposure in mammals; most related studies have reported on young developing retina that have undergone months of dark rearing after birth [9, 17, 19].

In our experiments, three sets of data support the conclusion that anesthesia does not play a major role in this functional shift. First, light manipulations were performed in both awake (Fig 2) and anesthetized animals (Fig 3A–3C for the dark exposed rat, Fig 4 for the restoration by a light exposure): the ERG amplitude and peak latency shifts were similar. Second, the ERGs were recorded under similar anesthesia, and sometimes recorded and compared in the same animals (Figs 3, 4, 5 and 6), possible anesthesia-induced influence on ERG were minimal. Third, the reduction of ERG was not due to prolonged anesthesia, since the ERG suppression also occurred in acutely anesthetized rats that had been dark exposed for 24 hours in a non-anesthetized state (Fig 2).

The shift on ERG b-wave in our result is different from classical dark adaptation. In a standard light intensity-ERG response curve, when ERG amplitude increases, it is reasonable that the peak latency decreases [26, 33], which is also shown in Fig 6C (blue trace). However, in the classical dark adaptation process, it is not the case: when ERG amplitude increases, the peak latency also increases [26]. This phenomenon is thus addressed as a unique property of dark adaptation. While in the 24 hours dark exposure-induced ERG suppression, the ERG amplitude shifted in the opposite way. In detail, it was not an expected extension of a further enhancement, but a gradual amplitude decline. Interestingly, accompanied with ERG amplitude decline, the peak latency also decreased, again different from the standard intensity-response curve. These unique properties support a new retinal mechanism for ERG suppression elicited by prolonged dark exposure, and further studies are needed to elucidate its mechanism.

Several aspects of our protocol resulted in the finding of a significant reduction in ERG b-waves in the 24 hours’ dark exposed rodents. First, after dark exposure began, the animal was transferred, anesthetized, prepared, and recorded in complete darkness with the aid of infrared night device. Second, a short 50 ms dim flash was employed during the recording session, and the sampled rate (once per 5–30 minutes) was minimized. These special efforts were performed to reduce overall light exposure, which may trigger the recovery process. In fact, our results demonstrated that brief dim light exposure could trigger the restoration process (see Fig 4), and 3 repetitive, brief (10 minutes) light exposures nearly fully restored ERG amplitudes to normal levels (Fig 2). The brief and dim light exposure might elicit a recovery that could contaminate the otherwise significant decrease in ERG amplitude in dark exposed animals.

In summary, the visually evoked ERG is fairly susceptible to prior visual experience in rodents. The time scale for the decrease was nearly 24 hours, and the recovery process can be triggered in 10 minutes. This upstream dynamics in the retina will inevitably be transferred to downstream visual regions, such as LGN and visual cortex, thus it is necessary to dissect the inherited and local functional dynamics in upstream regions in future.

## Supporting Information

S1 Fig The ERG response evoked by the light from our infrared device. The device were power on and provided infrared light between the two red vertical lines. (A-C) Three cases of ERG response to 50ms infrared light. (D-F) Three cases of ERG responses to 100ms infrared light. (G-I) Three cases of ERG responses to 1000ms infrared light.
(TIF)

S2 Fig The ERG b-wave amplitudes of the prolonged dark exposed (Prolonged DE) rats after a 10 min,– 0.24 log cd/m^2^ light exposure (LE). (TIF)
