# Supplementary figures and images for: The Shift of ERG B-Wave Induced by Hours' Dark Exposure in Rodents
Source: PLoS One. 2016 Aug 12;11(8):e0161010. doi: 10.1371/journal.pone.0161010 (PMC4982619; doi:10.1371/journal.pone.0161010)

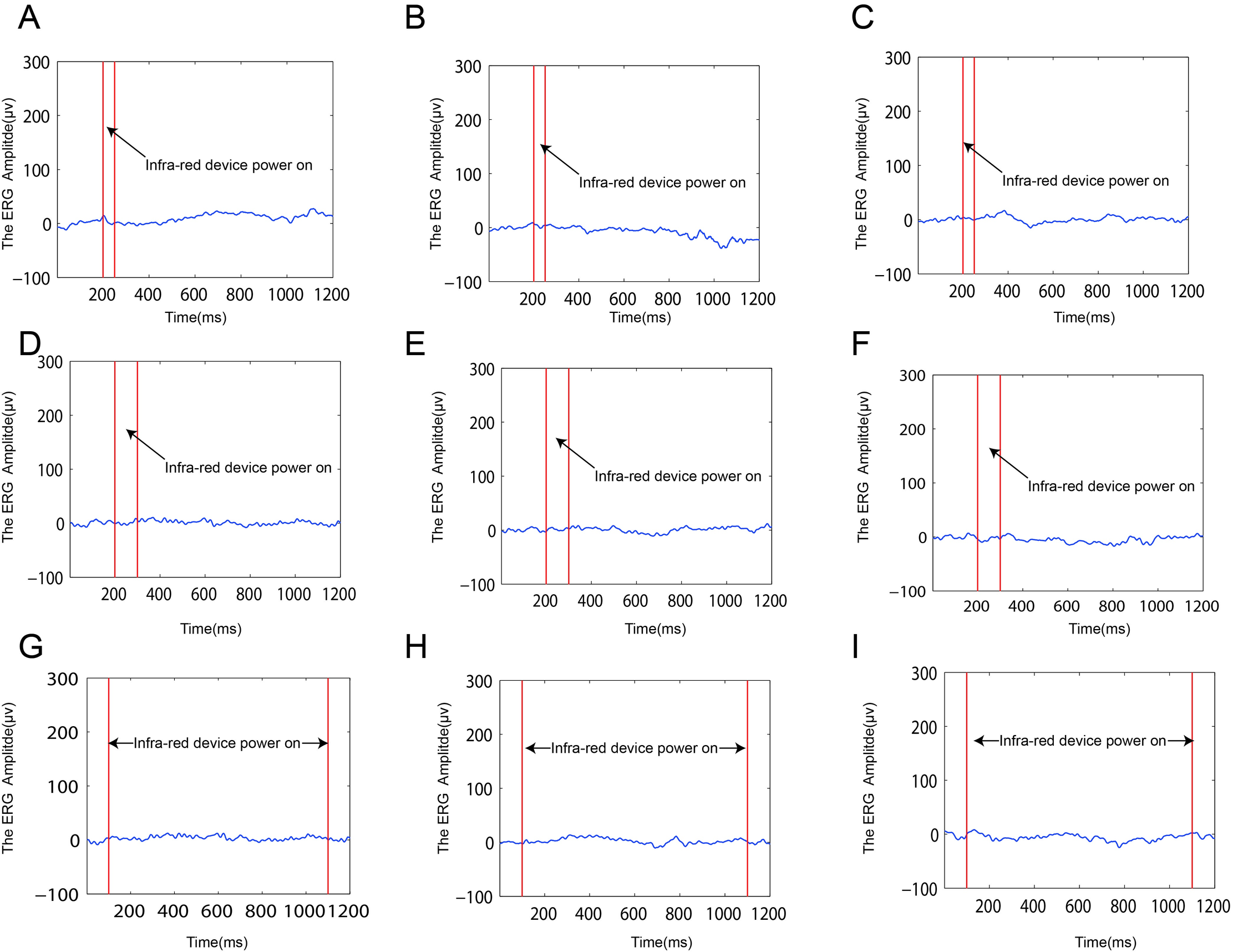

Supplement: S1 Fig — The device were power on and provided infrared light between the two red vertical lines. (A-C) Three cases of ERG response to 50ms infrared light. (D-F) Three cases of ERG responses to 100ms infrared light. (G-I) Three cases of ERG responses to 1000ms infrared light. (TIF) [file pone.0161010.s001.tif]

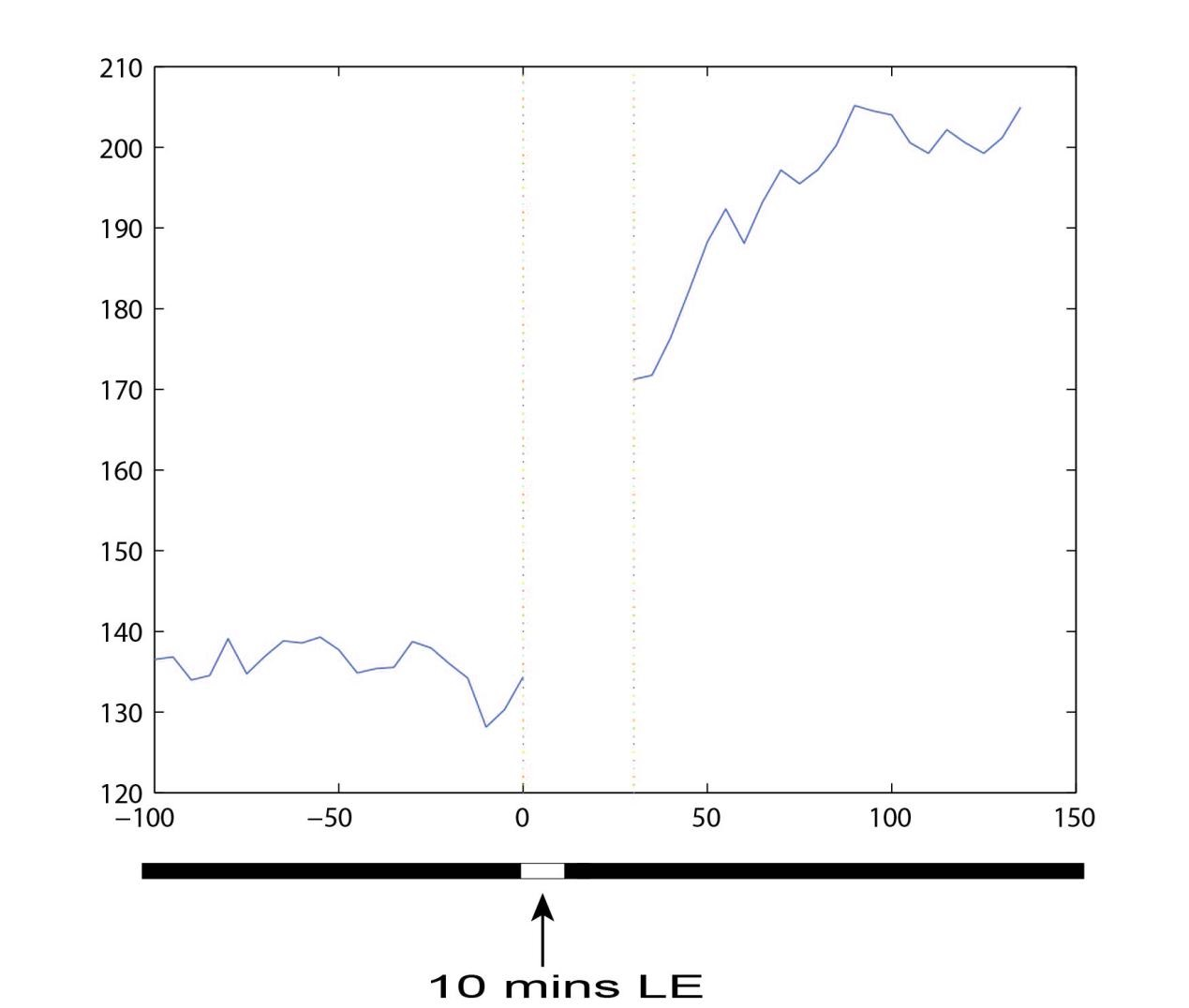

Supplement: S2 Fig — (TIF) [file pone.0161010.s002.tif]
